# Improved Productivity of Naringin Oleate with Flavonoid and Fatty Acid by Efficient Enzymatic Esterification

**DOI:** 10.3390/antiox11020242

**Published:** 2022-01-27

**Authors:** Jonghwa Lee, Kyeonga Kim, Jemin Son, Hyeseon Lee, Jin Han Song, Taek Lee, Heungbae Jeon, Hyun Soo Kim, Si Jae Park, Hah Young Yoo, Chulhwan Park

**Affiliations:** 1 Department of Chemical Engineering, Kwangwoon University, Seoul 01897, Korea; leejh9512@naver.com (J.L.); chzh3150@naver.com (K.K.); wkqh14@gmail.com (J.S.); d_b0225@naver.com (H.L.); jhssong75@paran.com (J.H.S.); tlee@kw.ac.kr (T.L.); 2 Department of Chemistry, Kwangwoon University, Seoul 01897, Korea; hbj@kw.ac.kr; 3 Department of Electronic Engineering, Kwangwoon University, Seoul 01897, Korea; hyunsookim@kw.ac.kr; 4 Division of Chemical Engineering and Materials Science, Ewha Womans University, Seoul 03760, Korea; 5 Department of Biotechnology, Sangmyung University, Seoul 03016, Korea

**Keywords:** antioxidant, flavonoid, naringin, flavonoid ester, flavonoid oleate, lipase, esterification, enzymatic synthesis, optimization

## Abstract

Naringin is a flavonoid found in citrus fruits. It exhibits biological activities, such as anticancer and antioxidant effects, but it suffers from low solubility and low stability in lipophilic systems. These drawbacks lead to difficulties in the commercial application of naringin, but they can be overcome through esterification. In this study, naringin oleate was synthesized by enzymatic esterification and optimal conditions for the reaction were investigated. Experiments were conducted focusing on the following parameters: enzyme type, enzyme concentration, molar ratio of naringin to oleic acid, reaction temperature, and reaction solvent. We further confirmed the degree of esterification based on the difference in the initial and the final naringin concentrations. A conversion of 93.10% was obtained under optimized conditions (Lipozyme TL IM 10 g/L, molar ratio 1:20, reaction temperature 40 °C, acetonitrile as solvent, and 48 h reaction time). Thus, naringin oleate, a high value-added material that overcomes the low hydrophobicity of naringin and enhances its performance, was obtained through esterification of naringin using oleic acid. This study presented a method for the efficient enzymatic synthesis that could ensure high conversion within a shorter reaction time compared with that required in previously reported methods.

## 1. Introduction

Flavonoids are phenolic secondary metabolites present in various plants and more than 8000 flavonoid forms have been discovered to date [1,2,3,4]. Studies have shown that flavonoids exert antioxidant [1,2,3], anti-inflammatory [4], and antitumoral [5] effects, and can reduce the risk of diabetes [6] and the incidence of cardiovascular disease [7].

Most flavonoids exist in nature in the glycosylated form. Glycosylated flavonoids contain various OH groups and, owing to their structure, most flavonoids show low solubility in lipophilic solvent systems [4,8]. Hydrophobicity is an important factor in cell membrane interactions and low hydrophobicity limits the absorption and interaction of flavonoids in the body, hindering their efficacy. Therefore, it is necessary to enhance the hydrophobic properties of flavonoids through acylation with aromatic and aliphatic compounds to overcome these limitations.

Acylation of flavonoids is usually carried out through esterification and transesterification reactions. Acyl donor or a substance having a carboxyl group, such as saturated/unsaturated fatty acid, dicarboxylic acid, or ester, may be used. The type of reaction carried out may differ depending on the type of acyl donor. Flavonoid esters produced through acylation have higher stability and higher solubility in lipophilic systems than the original flavonoids and some flavonoids gain higher physiological efficacy [4,9]. Li et al. [10] synthesized naringin esters with naringin and acyl donors which have various chain lengths. They determined the absorptivity of naringin esters in human intestinal epithelial Caco-2 cells, demonstrating that naringin octanoate that approximately 1.92-fold improved absorptivity than that of naringin. In addition, the free radical scavenging activity of naringin and naringin esters was compared through analysis of 1,1-diphenyl-2-picrylhydrazyl (DPPH) and 2,2′-azinobis (3-ethylbenzothiazoline-6-sulfonic acid) (ABTS). Relative to naringin, the free radical scavenging activities of naringin myristate by DPPH and ABTS were improved by approximately 1.8-fold, and 2.5-fold, respectively, compared with those of naringin. Flavonoid esters with improved absorptivity in cells and antioxidant activity exhibit improved application to the lipophilic matrix, thereby increasing the bioavailability.

**Figure 1:**
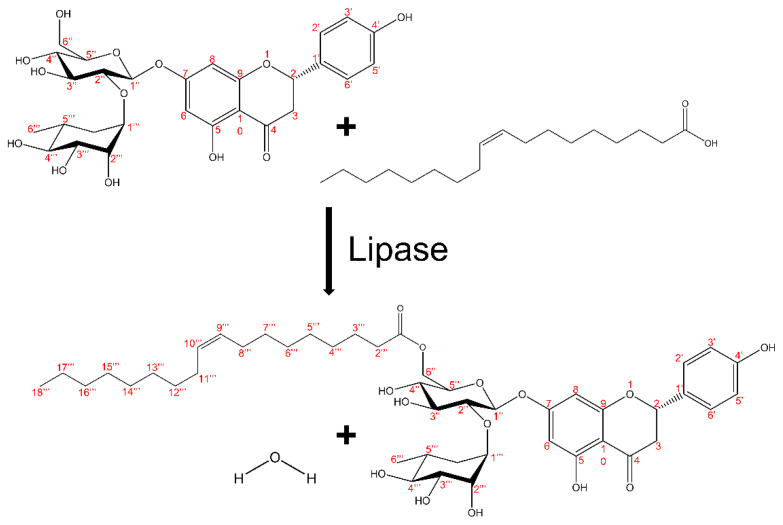
Regioselectivity of enzymatic naringin esterification.

The esterification reaction can be carried out both chemically and enzymatically. However, the chemical method requires a high temperature and high pressure, which can affect the phenolic structure of flavonoids [4,11,12,13]. In addition, chemical esterification can produce byproducts such as isomers, owing to its low regioselectivity [12,13,14,15]. However, in an enzymatic reaction, owing to high regioselectivity, the reaction occurs only at a specific reaction site. With regard to the naringin esterification reaction, it has been reported that the reaction occurs only at the 6″-OH position of several OH groups (Figure 1) [16]. Therefore, the enzymatic reactions can minimize the formation of isomers due to chemo-, enantio-, and regio-selectivity; moreover, unlike chemical reactions that require high temperatures and high pressure, enzymatic reactions can be carried out under mild reaction conditions [17]. However, enzymes incur a higher cost than chemical methods, are less stable, and are difficult to separate, which translate to poor process applicability. To minimize these issues, immobilized enzymes are used, which have higher stability and higher durability than free enzymes and they can be applied to various reactions and processes owing to the easy separation involved and the possibility to reuse the enzymes [12,13,15,18,19].

Owing to its broad substrate specificity and high regioselectivity, Lipase (E.C. 3.1.1.3) is widely used in the industry and in research. Lipase hydrolyzes lipids to produce fatty acids and glycerol and it catalyzes esterification and transesterification reactions under anhydrous conditions. The esterification reaction by lipase is accomplished by a ping-pong mechanism [4,12,13,15,20].

Naringin is a flavonoid contained in citrus fruits that is responsible for their bitter taste. It has a chemical structure in which two sugars are bound to naringenin. It is used in various products, such as supplements and cosmetics, because of its superior antioxidant and anticancer properties [4,21]. Citrus fruits are among the most popular fruits worldwide; however, they cause environmental problems owing to the amount of waste peel produced as result of their consumption [3,4,22]. Therefore, environmental and economic advantages are gained if the naringin contained in waste peels is used to produce high value-added products.

The number of studies on the esterification of flavonoids has been increasing due to interest in the efficacy and utilization of these compounds (Table 1). However, previous studies on naringin ester have been synthesis-oriented and the reactions reported involve long reaction times or low conversion. Besides, few studies have derived optimal values by analyzing the influence of key parameters in the esterification reaction of naringin and oleic acid. In the present study, the type of immobilized enzyme, concentration of enzyme, concentration of the substrate, reaction temperature, and reaction solvent were manipulated to analyze the effect of each parameter and to establish the optimal conditions for the enzymatic synthesis of naringin oleate.

## 2. Materials and Methods

### 2.1. Materials

Naringin (95%) and ethyl acetate (99.8%) were purchased from Sigma-Aldrich (St. Louis, MO, USA). Novozym 435 (immobilized *Candida antarctica* lipase B), Lipozyme TL IM (immobilized *Thermomyces lanuginosus*), and Lipozyme RM IM (immobilized *Rhizomucor miehei*) were purchased from Novo Nordisk Bioindustry (Bagsvaerd, Denmark). Dimethyl sulfoxide (DMSO) (99.5%), 1,4-dioxane (99.5%), acetone (99.5%), *tert*-butanol (99.5%), *tert*-amyl alcohol (99%), 1,2-dichloroethane (99%), isooctane (98%), oleic acid (99%), and molecular sieves (3-Å) were purchased from Dae-Jung Chemical and Metals Co. Ltd. (Gyunggido, Korea). Acetonitrile (99.5%) and tetrahydrofuran (THF) (99.5%) were purchased from Junsei Chemical Co. Ltd. (Tokyo, Japan). Methanol, water, and acetic acid (HPLC grade) were purchased from J.T. Baker (Phillipsburg, NJ, USA).

### 2.2. Enzymatic Esterification Condition

The enzymatic esterification of naringin and oleic acid was performed in a 50 mL serum bottle with a 20 mL working volume. The reaction proceeded for 48 h and the stirring speed (180 rpm) and reaction temperature were kept constant using a shaking incubator (Jeio Tech SI-600R, Daejeon, Korea). All experiments were conducted more than twice. The solvent used in the reaction was dried for 5 days with 150 g/L molecular sieves before use and naringin was also dried for 5 days in a desiccator before use. Molecular sieves used for drying were dried in an oven at 110 °C for at least 3 h before use.

### 2.3. Optimization of Reaction Condition

**Figure 2:**
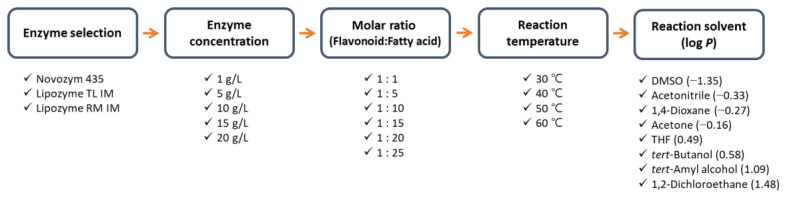
Optimization procedure for naringin oleate synthesis.

To find the optimal conditions for the enzymatic synthesis of naringin oleate, the following were manipulated in the stated order: enzyme type, enzyme concentration, molar ratio of the substrate, reaction temperature, and reaction solvent (Figure 2).

First, an experiment was conducted to determine the effect of the enzyme type. The experiment was conducted by selecting three types of immobilized enzymes (Novozym 435, Lipozyme TL IM, and Lipozyme RM IM) that are widely used commercially. Each enzyme was loaded at 5 g/L and the concentration ratio of naringin to oleic acid was 1:1 (10 mM). The reaction temperature was maintained at 50 °C and *tert*-amyl alcohol was used as the reaction solvent.

Second, different concentrations of the selected enzyme (Lipozyme TL IM) were used (1, 5, 10, 15, and 20 g/L) to investigate the effect of the enzyme concentration. The concentration ratio of naringin and oleic acid was 1:1 (10 mM). The reaction temperature was maintained at 50 °C and *tert*-amyl alcohol was used as the reaction solvent.

Third, to analyze the effect of the molar ratio of the substrate on the reaction, the experiment was conducted by changing the molar ratio of flavonoids to fatty acids to 1:1 1:5, 1:10, 1:15, 1:20, and 1:25. The experiment was carried out using Lipozyme TL IM (10 g/L) with a reaction temperature of 50 °C, and *tert*-amyl alcohol was used as the reaction solvent.

In the next step, to analyze the effect of temperature on the reaction, the reaction temperature was changed to 30, 40, 50, and 60 °C. The experiment was carried out using Lipozyme TL IM (10 g/L), at a molar ratio of 1:20, and *tert*-amyl alcohol was used as the reaction solvent.

Finally, to analyze the effect of the solvent on the reaction, various commercial organic solvents were investigated. DMSO, acetonitrile, 1,4-dioxane, acetone, THF, *tert*-butanol, *tert*-amyl alcohol, and 1,2-dichloroethane were used as the reaction solvents. The experiment was carried out using Lipozyme TL IM 10 g/L, molar ratio 1:20, reaction temperature 40 °C.

After finding the optimal experimental conditions, a time profile was created to determine the time required to achieve the highest conversion under the optimal conditions.

### 2.4. HPLC and FT-IR Analysis

To analyze the esterification reaction of naringin and oleic acid, 1 mL of a sample was separated from the enzyme through a syringe. The separated sample was diluted 10 times in methanol and filtered through a syringe filter (Advantec, DISMIC-13HP, Tokyo, Japan). The filtered sample was analyzed through HPLC (Agilent, 1260 Infinity II, Santa Clara, CA, USA) with a C18 column (5 µm, 4.6 × 250 mm, Youngjin Biochrom, Gyunggido, Korea). The injection volume was 5 µL, the flow rate was 1 mL/min, the column temperature was 50 °C, and the measurement was performed at a wavelength of 280 nm. Analytical components were separated according to the following concentration gradient: water contained 3% acetic acid (A), methanol (B), 0 min (70% A, 30% B), 5 min (0% A, 100% B), 10 min (0% A, 100% B), 15 min (70% A, 30% B), 20 min (70% A, 30% B). The conversion was calculated from the ratio of the initial naringin concentration to the naringin oleate concentration produced after the reaction [4].

(1) $$Conversion\;\left(\%\right)=\frac{Naringin\;oleate\;concentration}{Initial\;naringin\;concentration}\times 100\;\left(\%\right)$$

After the reaction was filtered to separate the immobilized enzyme, the solution was dried in a vacuum desiccator (Gast Manufacturing, Fair Plain, MI, USA). After drying, the powder was dissolved in a 30 mL (4:1 *v*/*v*) solution of isooctane/water at 45 °C and the isooctane phase was separated to remove fatty acids remaining in the reaction solution. This process was repeated twice. Then, the water phase was dried and dissolved in 40 mL (1:5 *v*/*v*) of ethyl acetate/water at 60 °C. The ethyl acetate layer was separated and dried to obtain naringin oleate. The purified naringin oleate was analyzed through FT-IR (JASCO, FT/IR-4600, Tokyo, Japan) and compared with the main peaks of naringin and oleic acid.

## 3. Results and Discussion

### 3.1. Effect of Enzyme Type on the Conversion of Naringin Oleate

The esterification reaction of naringin and oleic acid was carried out using an immobilized enzyme. Experiments were performed using Novozym 435 (immobilized *Candida antarctica* lipase B), Lipozyme TL IM (immobilized *Thermomyces lanuginosus*), and Lipozyme RM IM (immobilized *Rhizomucor miehei*) (Table 2). Conversion of 7.45%, 9.18%, and 6.23% were obtained for each enzyme and Lipozyme TL IM showed a higher conversion than the other two enzymes.

Lipase has a water layer around it that stabilizes the three-dimensional structure of the enzyme and maintains the polarity of the active site. When water activity is low, insufficient water is provided to form the essential water layer around the enzyme, which negatively affects enzymatic activity. High water activity can cause an excessively thick water layer, which leads to a competition effect between esterification and hydrolysis, also resulting in inhibition of the enzymatic activity [4,12,13,15,21,29,30].

Water is produced as a result of the esterification reaction. In the case of Novozym 435, the conversion is lower than that of Lipozyme TL IM, because the inhibitory effect of water is higher than that of Lipozyme TL IM [31]. In addition, Lipozyme RM IM is thought to have a low conversion because it has an inhibitory effect on enzyme activity in the presence of alcohol [32]. Lipozyme TL IM has been reported to show high conversion in the esterification reactions of flavonoids and phenols [31,33]. Lipozyme TL IM is also 8–10 times cheaper than Novozym 435 or Lipozyme RM IM [34].

Taking this into account, Lipozyme TL IM was expected to provide higher conversion and cost-efficiency esterification reactions. Therefore, Lipozyme TL IM was chosen as the appropriate enzyme.

### 3.2. Effect of Enzyme Concentration on the Conversion of Naringin Oleate

**Figure 3:**
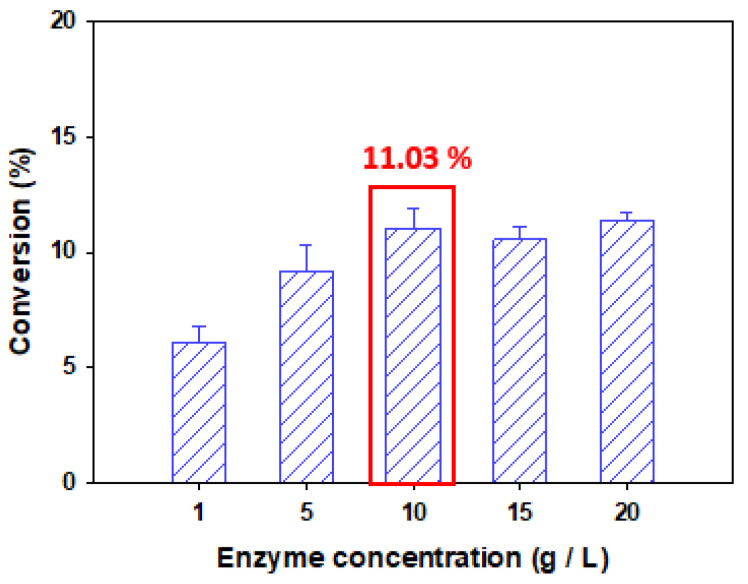
Conversion of naringin oleate influenced by enzyme concentration. (Lipozyme TL IM, 1:1 molar ratio of naringin to oleic acid, reaction temperature of 50 °C, *tert*-amyl alcohol as the solvent, reaction time of 48 h).

To analyze the effect of enzyme concentration on the conversion, the experiment was conducted by using different concentrations of Lipozyme TL IM 1 g/L, 5 g/L, 10 g/L, 15 g/L, and 20 g/L. Conversion of 6.08%, 9.18%, 11.03%, 10.54%, and 11.36% were obtained for these concentrations, respectively (Figure 3). The lowest conversion was obtained at a concentration of 1 g/L, and the conversion tended to increase as the enzyme concentration increased to 10 g/L. However, the conversion did not increase significantly at enzyme concentrations above 10 g/L.

When sufficient substrate is present in the enzyme reaction, the concentration of the enzyme-substrate intermediate increases as the enzyme concentration increases and the reaction rate increases according to the Michaelis–Menten equation [4,12,13,35]. The active sites of the enzyme molecules, which are present in excess, are not sufficiently exposed to the substrate and thus do not contribute to the reaction [15,21,36]. In addition, excessive enzyme concentrations cause a limitation of the mass transfer of substrates and products [12,13,29]. Another explanation is acids and alcohols bind to enzymes competitively. Acids can preferentially bind to enzymes at low enzyme concentrations. However, at high enzyme concentrations, alcohols can bind to the enzyme and have a competitive inhibitory effect. Therefore, an increase in excessive enzyme concentration does not lead to an increase in conversion owing to competitive inhibition [13,15,37].

For this reason, it was determined that an increase in the enzyme concentration above 10 g/L did not have a significant effect on the conversion; thus, the optimal enzyme concentration was set to 10 g/L.

### 3.3. Effect of Molar Ratio on the Conversion of Naringin Oleate

**Figure 4:**
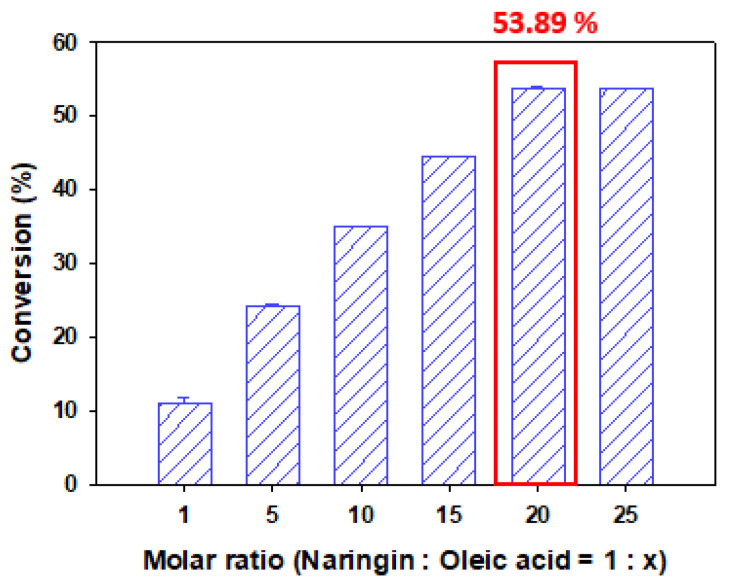
Conversion of naringin oleate influenced by molar ratio between naringin and oleic acid. (10 g/L of TL IM, reaction temperature of 50 °C, *tert*-amyl alcohol as the solvent, reaction time of 48 h).

The theoretical molar ratio for reaction between naringin and oleic acid is 1:1. However, a high reaction rate can be achieved within a short period if an excessive number of reactants are used. Naringin shows low solubility in organic solvents, owing to its high hydrophilicity. Therefore, the concentration of oleic acid was changed to 1:1, 1:5, 1:10, 1:15, 1:20, and 1:25 against naringin. Conversion of 11.03%, 24.12%, 34.92%, 44.45%, 53.89%, and 53.74% were obtained, respectively (Figure 4). The conversion tended to increase with an increase in the amount of oleic acid to 1:20.

This is because the thermodynamic equilibrium shifts in a direction favorable to the formation of naringin ester, owing to the increase in oleic acid concentration [4,37]. Previous studies have shown that conversion increases with an increase in the molar ratio of the substrate [12,13,15,32,38,39]. However, at a molar ratio of 1:25, the conversion was 53.74%, which was slightly less than that at 1:20. The reason is that excessive substrate concentration negatively affects the conversion. Excessive concentration of the substrate increases the viscosity of the solvent, interferes with mass transfer, changes the polarity of the solvent, affects the activity of the enzyme, and causes the substrate splitting effect [12,13,29,40,41].

Therefore, a molar ratio of 1:20 was considered the optimal ratio, which is the highest molar ratio not to exert an inhibitory effect on the reaction.

### 3.4. Effect of Reaction Temperature on the Conversion of Naringin Oleate

**Figure 5:**
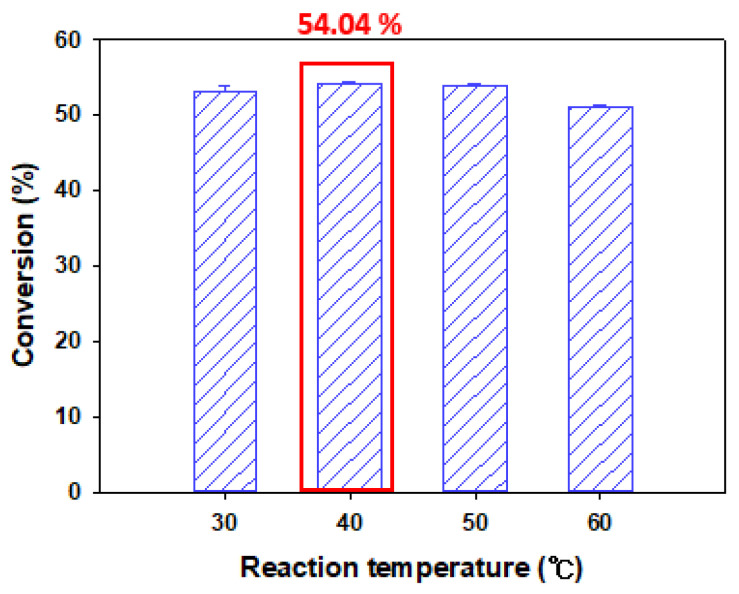
Conversion of naringin oleate influenced by reaction temperature. (10 g/L of TL IM, 1:20 molar ratio of naringin to oleic acid, *tert*-amyl alcohol as the solvent, reaction time of 48 h).

Reaction temperature has various effects on enzymatic reactions. Therefore, to investigate the effect of reaction temperature on naringin oleate synthesis, experiments were conducted using reaction temperatures of 30, 40, 50, and 60 °C. Conversion of 53.25%, 54.04%, 53.89%, and 50.99% were obtained, respectively (Figure 5). At a reaction temperature of 40 °C, the highest conversion of 54.04% was obtained, but there was no significant difference overall.

Since the esterification reaction is endothermic [12,13,39], the reaction rate increases with increases in temperature. Furthermore, when the temperature increases, the viscosity of the reaction solvent decreases so that mass transfer between the enzyme and the substrate may occur more easily. However, an increase in temperature increases water activity and excessive water activity can affect enzyme activity [12,13,15]. It has also been reported that an excessive increase in temperature can cause irreversible denaturation of the enzyme [42]. Noel and Combes [43] reported that an increase in temperature above 40 °C caused a sharp decrease in enzyme activity.

Therefore, a reaction temperature of 40 °C was considered the optimal temperature.

### 3.5. Effect of Solvent Type on the Conversion of Naringin Oleate

The role of the solvent in enzymatic reaction is also important. The factors affecting the conversion of solvents are very diverse, including solubility of the substrate, hydrophobicity, polarity, toxicity to enzymes, and regioselectivity [12,13,15,19,21]. Lipase can catalyze both esterification and hydrolysis reactions, and the reaction varies depending on the substrate and the surrounding environment. Anhydrous conditions catalyze the esterification reaction; however, a hydrolysis reaction can occur in the presence of moisture. Therefore, if an organic solvent under anhydrous conditions is used, high activity and stability of the lipase can be obtained [44].

Commonly used organic solvents such as DMSO, acetonitrile, 1,4-dioxane, acetone, THF, *tert*-butanol, *tert*-amyl alcohol, and 1,2-dichloroethane were used for the experiment. The conversion using these solvents was 18.47%, 93.10%, 19.22%, 26.68%, 17.33%, 45.65%, 54.04%, and 16.34%, respectively.

Lipase is known to exhibit high activity in hydrophobic solvents [15,45]. However, glycosylated flavonoids such as naringin have low solubility in hydrophobic solvents, making it difficult to react [4,46]. The effect of the solvent on the conversion depends on the characteristics of flavonoids and fatty acids. Therefore, it is necessary to find a solvent that can maintain adequate enzymatic activity while having high solubility for polar flavonoids and non-polar fatty acids [9,15].

Hazarika et al. [47] demonstrated that the initial reaction rate of lipase becomes faster in a hydrophobic solvent becomes faster as it has a lower log *P* value and higher polarity. Log *P* is the logarithm of the concentration ratio when water and octanol are mixed. In general, if the log *P* value is less than 2, the solvent is hydrophilic, and if the log *P* value is higher than 4, the solvent is hydrophobic [12]. The dielectric constant is a measure of the ability of a solvent to separate opposite charges from each other and a higher dielectric constant results in a higher polarity. The log *P* values and dielectric constant values of the solvents used in the experiment are shown in the table below (Table 3).

Acetone and acetonitrile have low log *P* values and high dielectric constants and they show a high conversion in the esterification reactions of many flavonoids [9]. Acetonitrile, selected as the optimal solvent in this experiment, has a log *P* value of –0.33 and a high dielectric constant of 37.5. Chebil et al. [48] and Li et al. [49] also showed a high conversion using acetonitrile. Among the solvents used, DMSO had the lowest log *P* value and a high dielectric constant, but the conversion was found to be low, which may be due to the inactivation of enzymes under DMSO conditions [50].

**Figure 6:**
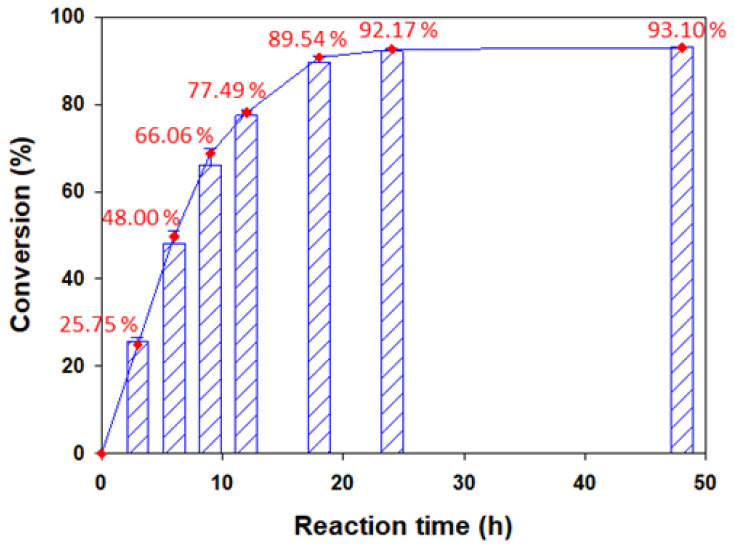
Conversion of naringin oleate as a function of reaction time. (10 g/L of Lipozyme TL IM, 1:20 molar ratio of naringin to oleic acid, reaction temperature of 40 °C, acetonitrile as the solvent, reaction time of 48 h).

After completing the solvent selection step, experiments were conducted to determine the time to reach maximum conversion under optimal reaction conditions (Lipozyme TL IM 10 g/L, molar ratio 1:20, reaction temperature 40 °C, acetonitrile). Conversion of 25.75%, 48.00%, 66.06%, 77.49%, 89.54%, 92.17%, and 93.10% were obtained at 3 h, 6 h, 9 h, 12 h, 18 h, 24 h, and 48 h, respectively (Figure 6). The results show that conversion increased only 0.93% during the reaction time from 24 to 48 h and, therefore, that the reaction was completed within 48 h. This reaction time is shorter than that of previous studies.

### 3.6. FT-IR Analysis

**Figure 7:**
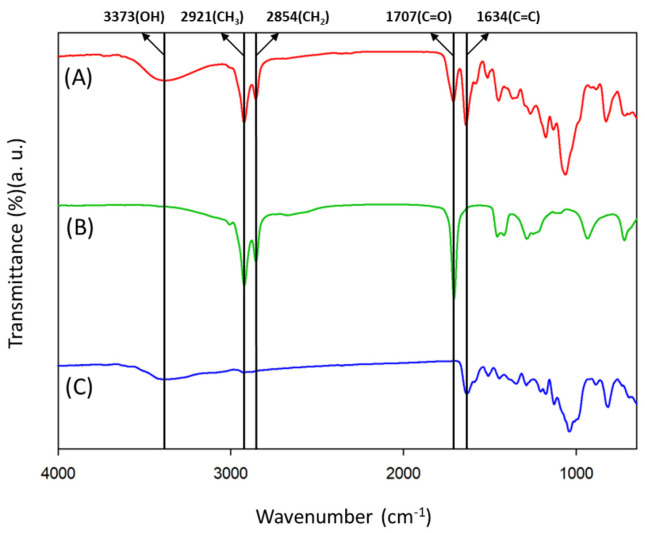
FT-IR spectra of naringin oleate synthesis (**A**) naringin oleate, (**B**) oleic acid, (**C**) naringin.

FT-IR analysis was performed, in the wavelength range of 650–4000 cm^−1^, to analyze naringin, oleic acid, and naringin oleate (Figure 7). The spectrum for naringin (C) showed a peak owing to the -OH functional group in the region of 3373 cm^−1^ and a peak indicating C=C bond at 1634 cm^−1^. In the oleic acid spectrum (B), distinguished peaks appeared at 2921 cm^−1^, 2854 cm^−1^, and 1707 cm^−1^ because of -CH_3_, -CH_2_-, and C=O, respectively. In the naringin oleate spectrum (A), presence of both naringin and oleic acid functional groups could be detected, confirming the synthesis of naringin oleate [51].

**Figure 8:**
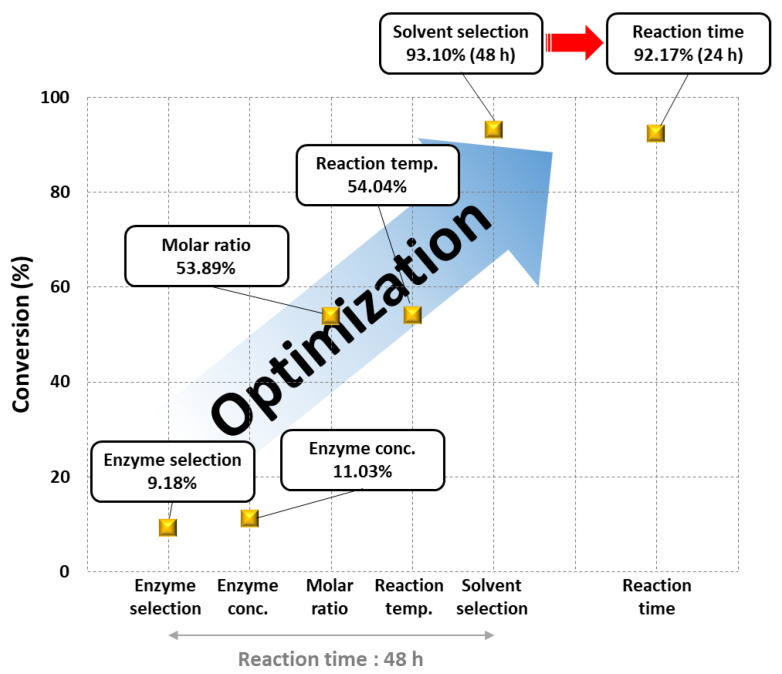
Improved conversion of naringin oleate by optimization.

In this study, we evaluated the effect of each reaction parameter on the synthesis of naringin oleate by optimization using the OFAT method. Figure 8 summarizes the improvement in conversion by optimization of each parameter, affecting the synthesis of naringin oleate. Among the five parameters, molar ratio and solvent type had a significant effect on the synthesis of naringin oleate. When the molar ratio and solvent type were optimized, the conversion increased significantly from 11.04% to 53.89% and from 54.04% to 93.10%, respectively. Overall, the conversion significantly increased from 9.18% to 93.10% within 48 h by optimization of the naringin oleate synthesis reaction conditions. We further confirmed the conversion according to the reaction time under the optimal conditions, demonstrating a conversion of 92.17% obtained within 24 h. This study focused on the optimization of naringin oleate synthesis and a separation and a purification steps were performed. The purified product was confirmed using FT-IR, which confirmed that naringin oleate was synthesized. In the next stage, a statistical analysis will be conducted to determine the correlation of each reaction parameter based on these studies.

## 4. Conclusions

Naringin oleate was synthesized by enzymatic esterification reaction of naringin and oleic acid and experiments were conducted by varying several parameters to determine the optimal reaction conditions. Optimal conditions for the synthesis of naringin oleate were Lipozyme TL IM 10 g/L, molar ratio 1:20, reaction temperature 40 °C, and acetonitrile as the reaction solvent, with the highest conversion of 93.10% for 48 h. A conversion of 92.17% was obtained under optimized conditions when the reaction time was 24 h. The synthesis of naringin oleate was confirmed by HPLC and FT-IR analysis. Each parameter has a substantial influence on enzymatic reactions, and it will be necessary to apply a statistical methodology to further shorten the reaction time and to analyze the correlations of each reaction parameter. This study confirmed the commercial potential of the process by achieving higher conversions within a shorter reaction time compared with that reported in previous studies. In addition, the current naringin oleate synthesis was conducted employing the commonly used immobilized enzyme, Novozym 435, but the experiment was conducted using the cost-effective Lipozyme TL IM. Therefore, the findings of this study are expected to contribute to the development of cost-effective processes that can potentially be commercialized.

## Figures and Tables

**Table 1:** Conversion of naringin ester synthesis using enzymes in previous studies.

Substrate		Reaction Condition	Conversion (%)	Reference
Flavonoid	Acyl Donor			
Naringin	Lauric acid	Novozym 435 10 g/L, 1:7, 60 °C, acetonitrile	36%	[23]
Naringin	Palmitic acid	Novozym 435 10 g/L, 1:5, 60 °C, *tert*-amyl alcohol, 55 h	43%	[24]
Naringin	Oleic acid	Novozym 435 15 g/L, 1:4, 50 °C, *tert*-amyl alcohol, 96 h	78%	[25]
Naringin	Sunflower oil	Novozym 435 10 g/L, 1:6, 65 °C, acetonitrile, 90 h	85%	[26]
Naringin	Oleic acid	Novozym 435 11 g/L, 1:5, 50 °C, acetone, 96 h	87%	[27]
Naringin	Oleic acid	Novozym 435 12 g/L, 1:4, 45 °C, acetonitrile, 96 h	88%	[28]
Naringin	Oleic acid	Lipozyme TL IM 10 g/L, 1:20, 40 °C, acetonitrile, 48 h	93.10%	This study

**Table 2:** Properties of commercial immobilized lipases and conversion of naringin oleate influenced by enzyme type. (5 g/L of enzyme concentration, 1:1 molar ratio of naringin to oleic acid, reaction temperature of 50 °C, *tert*-amyl alcohol as the solvent, reaction time of 48 h).

Enzyme	Source	Regioselectivity	Substrate Specificity	Support	Conversion (%)
Novozym 435	*Candida antarctica*	Non-specific	Esters and alcohols	Acrylic resin	7.45
Lipozyme TL IM	*Thermomyces lanuginosus*	1,3-specific	Esters	Silica gel	9.18
Lipozyme RM IM	*Rhizomucor miehei*	1,3-specific	Esters	Phenol-formaldehyde copolymer	6.23

**Table 3:** Properties of organic solvents and conversion of naringin oleate influenced by solvent. (10 g/L of TL IM, 1:20 molar ratio of naringin to oleic acid, reaction temperature of 40 °C, reaction time of 48 h).

Solvent	Log *P*	Dielectric Constant	Conversion (%)
DMSO	−1.35	46.7	18.47
Acetonitrile	−0.33	37.5	93.10
1,4-Dioxane	−0.27	2.2	19.22
Acetone	−0.16	20.7	26.68
THF	0.49	7.5	17.33
*tert*-Butanol	0.58	10.9	45.65
*tert*-Amyl alcohol	1.09	5.8	54.04
1,2-Dichloroethane	1.48	10.4	16.34

## Data Availability

The data is contained within the article.
